# Severity of Coronavirus Disease 2019 (COVID-19): Does Surfactant Matter?

**DOI:** 10.3389/fmicb.2020.01905

**Published:** 2020-08-26

**Authors:** Ralf Weiskirchen

**Affiliations:** Institute of Molecular Pathobiochemistry, Experimental Gene Therapy and Clinical Chemistry (IFMPEGKC), RWTH University Hospital Aachen, Aachen, Germany

**Keywords:** infection, surfactant, immunity, gender, body mass index, genetics, smoking, pandemic

## Introduction

Worldwide, the Coronavirus disease 2019 (COVID-19) pandemic has led to health and economic damage that cannot yet be accurately predicted. First clinical studies of hospitalized patients have shown that there are risk factors majorly contributing to the outcome of COVID-19 infection. However, a complete picture of disease pathogenesis and underlying mechanisms is still missing. Therefore, new theories that lead to a better understanding of COVID-19 are urgently needed. The understanding will lead to new diagnostic and of course therapeutic possibilities to target the severe acute respiratory syndrome coronavirus 2 (SARS-CoV-2). Here it is hypothesized that lowered concentrations or altered composition of pulmonary surfactant is a critical risk factor for COVID-19.

## Discussion

As we continue to learn more about the virus, there are still many key questions that scientists are urgently try to answer:

1. *Age*: Children seem to be a less susceptible to COVID-19 and disease severity at young age is lower than in adults (Lee et al., 2020). Why?
2. *Obesity/diabetes*: Obesity (high body mass index) and diabetes seems to be a risk factor for poor adverse outcomes of COVID-19 (Malavazos et al., 2020). Why?
3. *Smoking*: The estimated prevalence of current smoking among hospitalized patients with COVID-19 is rather low (Farsalinos et al., 2020a). Why?
4. *Blood pressure*: Hypertension and intake angiotensin converting enzyme (ACE) inhibitors and angiotensin receptor blockers (ARBs) increase risk of developing severe and fatal COVID-19 (Fang et al., 2020). Why?
5. *Coagulation*: Hypercoagulability is associated with disease severity (Liu et al., 2020). Why?
6. *Gender*: Men with COVID-19 are more at risk for worse outcomes and death (Jin et al., 2020). Why?
7. *Infection*: The coronavirus by no means affects all persons, some seems to be resistant. Why?
8. *Immunity*: Do COVID-19 patients develop long-term immunity?
9. *Diagnosis*: The presence of virus in patients is verified in sputum, salvia, or a swab from deep in the nasal passages using antigen-based methods and RT-PCR tests. Serological tests are used to demonstrate previous infections with SARS-CoV-2. Can diagnosis be improved and/or simplified?
10. *Therapy*: Several vaccines, neutralizing antibodies and drugs are in the pipeline for COVID-19 therapy. Are there better alternatives?

Experts from all over the world are presently discussing these topics and a number of plausible theories arose. In regard to age, suggested reasons are a more active innate immune response, healthier respiratory tracts, differences in the distribution/maturation/function of viral receptors, fewer outdoor activities and less international travel making younger persons less likely to contract the virus (Lee et al., 2020). The propensity of people with obesity to develop more serious complications if exposed to a virus is attributed to multiple factors, such as a chronic inflammatory status and delayed and ineffective immune response (Malavazos et al., 2020). Regarding smoking, a preliminary hypothesis assumes that the virus enters the body through neurons of the olfactory systems. In this scenario, nicotine inhibits the respective receptor (nAChR) explaining the low frequencies of smokers in the COVID-19 cohorts and suspecting nicotine or other pharmaceutical nicotine products as therapeutic agents to target COVID-19 (Farsalinos et al., 2020b; Kloc et al., 2020).

It is suggested that there is a complex interplay between COVID-19 and the renin-angiotensin-aldosterone system (RAS), in which SARS-CoV-2 has a spike protein receptor-binding domain with high affinity for human angiotensin-converting enzyme 2 (ACE2) (Andersen et al., 2020), which regulate both the cross-species and human-to-human transmission of the virus. The virus likely recognizes ACE2 orthologous from many other species, except mouse and rat (Wan et al., 2020). It was found that the expression of this receptor is very low in healthy young persons, while it increases in patients with vascular diseases (Singh et al., 2020). ACE and ARBs increase expression of the metallopeptidase ACE2, which is expressed primarily in alveolar epithelial type II cells and serves as a viral reservoir and functional entry receptor of SARS-CoV (Li et al., 2003). Therefore, these drugs were proposed to increase viral uptake and risk of developing severe and fatal COVID-19 (Fang et al., 2020). In line, it was proposed that the SARS-CoV-2 spike protein activates RAS in the lung, thereby promoting platelet adhesion and aggregation and increasing the risk for pulmonary embolism, hypertension and fibrosis. On the contrary, anticoagulation agents improve the clinical outcome of COVID-19 (Liu et al., 2020).

The risk factor “male gender” and the higher dying rate of men in COVID-19 is supposed to be consequence of the general demographic fact of a general shorter expectancy in men compared to women (Jin et al., 2020). However, there is already clear indication that there exist sex differences in surfactant biosynthesis putatively be ascribed to the stimulating effects of estrogens. This “male disadvantage” has been recognized clinically and in many experimental models (Torday and Nielsen, 1987; McCoy et al., 1999).

Genetic host resistance to coronaviruses is assumed to occur at three levels, namely genetic control of the level of cellular entry receptors, genetic control at the level of macrophage activity, and genetic control at the level of acquired immunity (Buschman and Skamene, 1995). Resistance as a host response to coronavirus infection is predominantly mediated by adaptive immunity comprising humoral and cell-mediated immunity (Li et al., 2020). A different susceptibility to COVID-19 might therefore be evoked by many factors related to the innate or adaptive immune system. It should be mentioned, that the human population has a small pre-existing immunity against SARS-CoV-2 and the T cell response to the structural proteins S, M, and N of the SARS virus are long-lasting and persistent (Grifoni et al., 2020). However, these findings do not mean that persons that were infected are protected from reinfection.

Reliable laboratory diagnostic for COVID-19 is required to prevent false negative and positive results which both have fatal consequences. Presently, RT-PCR is used as a diagnostic tool using nasal swab, tracheal aspirate or bronchoalveolar lavage (BAL) samples. However, they don't provide data about disease outcome and have only an estimated sensitivity rate around 66–80% (Pascarella et al., 2020). Simple analysis of most common COVID19-related symptoms such as fever (98%), cough (77%), and dyspnea (63.5%) are suitable to estimate mortality of critically ill patients (Yang et al., 2020). Similarly, the combination of three biomarkers (lactic dehydrogenase, high-sensitivity C-reactive protein, and lymphocyte count) identified by machine learning tools were shown to predict the mortality of individual patients more than 10 days in advance with more than 90% accuracy, suggesting that the combination of clinical symptoms and biomarkers can reflect the entire spectrum of disease from the earliest manifestations to the terminal stages (Yan et al., 2020). Currently, there are no effective vaccine or approved specific antiviral COVID-19 agents available. However, convalescent plasma therapy was effective to achieve serum SARS-CoV-2 RNA negativity in severe COVID-19 patients (Duan et al., 2020). So there are already promising strategies to limit or delay the spread of COVID-19 in the near future. Certainly, increasing funding in the research area “Corona” will further drive discovery providing the basis for successful targeting SARS-CoV-2. Undoubtedly, biased or unbiased biomarker discovery will be one important piece in the puzzle to target the COVID-19 pandemic.

Although never working in the field of Corona, I would highlight several features of pulmonary surfactant rendering this surface-active lipoprotein complex relevant for the pathogenesis, diagnostics or therapy of COVID-19. Pulmonary surfactant is composed primarily of phospholipids and four surfactant-associated proteins, namely SP-A, SP-B, SP-C, and SP-D. This lipid-protein complex is essential for the biophysical function of the lung. It stabilizes the delicate structure of the mammalian alveoli along with successive compression-expansion respiratory cycles by reducing surface tension at the air-liquid interface (Autilio and Pérez-Gil, 2019). In addition, this mixture of lipids and proteins constitute a first barrier to the access of pathogens to the rest of the organism via the large respiratory surface, preventing the dissemination of pathogens and modulating immune responses (Han and Mallampalli, 2015; Autilio and Pérez-Gil, 2019). In this regard, most important are SP-A and SP-D, which are members of a family of innate immune proteins termed collectins involved in bacterial and viral clearance (Han and Mallampalli, 2015). These collagen-containing C-type lectins further have ability to opsonize pathogens and facilitate their phagocytosis by cells of the innate immune system, such as macrophages and monocytes (Han and Mallampalli, 2015). Most important, a paper published already 13 years ago demonstrated that SP-D could bind to the SARS-CoV carbohydrate moieties presented on the S-protein of the virus, thereby preventing activation of macrophages and potentially preventing pulmonary inflammation suggesting that SP-D might independent of surfactant itself act as a COVID-19-related factor at the alveolar spaces (Leth-Larsen et al., 2007). Although the exact domains in the SARS-CoV S-protein interacting with SP-D were not dissected in the mentioned study and natural carbohydrate moiety of SARS-CoV-2 might be rather different, the study supports the view that deficiencies in collectins acting as host lectins will have significant impact on innate immunity. In line, SP-A deficient mice exhibited impaired clearance against viruses (e.g., RSV) and showed greater pulmonary infiltration with polymorphonuclear leukocytes (LeVine et al., 1999). Similarly, SP-A inhibited influenza A virus infection of lung epithelial cells (Al-Qahtani et al., 2019), while a recombinant fragment of human SP-D was sufficient to act as an entry inhibitor of influenza A virus *in vitro* (Al-Ahdal et al., 2018).

SP-A protein levels are increased in the serum, plasma and sputum of smokers (Kida et al., 1997; Nomori et al., 1998; Mazur et al., 2011), providing another rationale for the proposed beneficial effects of smoking or nicotine consumption in preventing COVID-19 (Farsalinos et al., 2020a). Furthermore, the composition of surfactant highly significant changes rapidly with exercise in a manner related to fitness and training (Doyle et al., 1994, 2000). In line, there is an inverse association between serum SP-D and body mass index (Zhao et al., 2007), and moreover inverse association with human obesity (Sorensen et al., 2006). Similarly, it was shown that SP-A levels negatively correlate with body mass index (Lugogo et al., 2018), possibly explaining why persons with high body mass index (BMI) having lower SP-A and SP-D levels are more susceptible to COVID-19.

In regard to age, it was shown that the quantities of alveolar and total SP-A to body weight and in relationship to alveolar surface area decrease significantly during aging in rats (Ohashi et al., 1994). In humans, the total SP-A serum levels are not affected by age (Nomori et al., 1998). However, human SP-A consists of two functional genes (SP-A1, SP-A2) and a gradual decrease in the SP-A1/SP-A ratio was observed in healthy subjects with increased age (Tagaram et al., 2007). Moreover, the total phospholipid concentration in bronchoalveolar lavage fluid was found higher in children <8 years of age and shown to decrease with age (Ratjen et al., 1996).

Historical experiments conducted in lambs and newborn piglets revealed that surfactant therapy results in a significant increase in pulmonary blood flow, decrease in pulmonary vascular resistance, reduction in right-to-left shunting, and decrease in mean arterial blood pressure (Moen et al., 1996; O'Toole et al., 1996). Conversely, it can be concluded that lowering surfactant compounds are causative involved in hypertension presenting a risk factor for developing severe and fatal COVID-19.

Collectively, lowered concentrations or altered composition of pulmonary surfactant might be a critical risk factor for COVID-19. Furthermore, it is well-known that polymorphisms in surfactant proteins can predispose individuals to community acquired pneumonia and/or acute respiratory distress syndrome (Lin et al., 2000; Gong et al., 2004). This provides another argument why surfactant proteins or surfactant constituents are critical factors contributing to COVID-19 susceptibility and outcome.

The potential connection of SARS-CoV-2 virus infection with a deficit in pulmonary surfactant or factors thereof and the administration of surfactant as a treatment modality for different phenotypes of COVID-19 are presently intensively discussed (Koumbourlis and Motoyama, 2020). Some authors speculate that the early administration of natural lung surfactant could improve the pulmonary function to restore pulmonary barrier function in patients with COVID-19 pneumonia, thereby reducing the duration of ventilation therapy and contributing to patients' recovery (Mirastschijski et al., 2020). Indeed, this could be easily done by adding the reconstituted lyophilizate powder into the trachea tube of the ventilated COVID-19 patient. In another hypothesis, it is argued that pulmonary or some chemical surfactants or stimulants of natural surfactant production may be effective not only for the treatment but also for the prophylactic protection for SARS-CoV-2 (Takano, 2020). In the respective theory, it is argued that the pulmonary surfactant not only is beneficial to reduce surface tension or to increase lung compliance, but also is a strong defender against the virus itself. However, none of the theories is actually supported with true experimental evidence. Even more, COVID-19 pathogenesis is rather complex consisting of three distinct phases classified on the site the respiratory epithelium infected (Mason, 2020). Consequently, the potential therapeutic outcome after surfactant treatment will strongly depend on the location (i.e., nose, conducting airways, alveoli) to which the virus has advanced.

Presently, there are no studies available analyzing COVID-19 susceptibility and surfactant biology. However, such studies will be of fundamental importance. If surfactant protects against COVID-19, supplementation after early SARS-CoV-2 infection with purified or recombinant forms of surfactant compounds will offer novel therapeutic avenues in controlling pulmonary infection during COVID-19. It would be desirable if the biomarker “surfactant” would be included in as many COVID-19 studies as possible and to assess the fate of endogenous surfactant in bronchoalveolar lavage material of patients in ongoing and future investigations or trials.

## Author Contributions

RW has drafted this contribution.

## Conflict of Interest

The author declares that the research was conducted in the absence of any commercial or financial relationships that could be construed as a potential conflict of interest.
